# Seed sojourn and fast viability loss constrain seedling production of a prominent riparian protection plant *Salix variegata* Franch

**DOI:** 10.1038/srep37312

**Published:** 2016-11-24

**Authors:** Qiaoli Ayi, Bo Zeng, Jianhui Liu, Shaohua Shi, Hangang Niu, Feng Lin, Yeyi Zhang

**Affiliations:** 1 Key Laboratory of Eco-environments in Three Gorges Reservoir Region (Ministry of Education), Key Laboratory of Plant Ecology and Resources Research in Three Gorges Reservoir Region, School of Life Sciences, Southwest University, Chongqing 400715, P. R. China

## Abstract

*Salix variegata* Franch, a prominent plant applied in riparian shelter vegetation in Three Gorges reservoir region of China, produces many seeds every year but generates only a few or no seedlings. Whether the low seedling production of *S. variegata* is caused by seed sterility or by rapid loss of seed viability remains unknown. We investigated the sojourn time of mature seeds in capsules produced in early, mid, and late reproductive season and the germinability of mature seeds fresh or stored after different period of time. The sojourn time of seeds in capsules was 2.89, 3.95, and 4.72 days in early, mid, and late reproductive season, respectively. The maximal germination percentage of non-stored fresh seeds produced in early, mid, and late reproductive season was 93.33%, 78.67%, and 40%, respectively, which indicates mature seeds were not sterile. The longest viability-retaining time of seeds produced in early, mid, and late reproductive season was only 8, 16, 16 days, respectively, indicating that mature seeds of *S. variegata* lost viability very rapidly. Mature seeds possessed good viability, but their rapid viability loss caused the low seedling production and hampered the population growth of *S. variegata* in the riparian area of Three Gorges reservoir region.

Three Gorges reservoir is the largest reservoir in China, which is also a large reservoir well-known in the world^1^,^2^,^3^,^4^,^5^. Three Gorges reservoir began its operation in 2003, since then, it has brought large benefits in flood control, electricity generation, and navigation^6^,^7^.

Three Gorges dam is built across Yangtze River, the dam construction results in the permanent submergence of the original riverbanks of Yangtze River within the reservoir, and leads to the formation of new banks around the reservoir consequently. In order to stabilize the new banks, alleviate the soil erosion of the newly formed riparian zone, reduce the influx of diffuse pollutants from surrounding farmlands into the reservoir so as to protect the water environment, Chinese central and local governments have implemented the construction of riparian shelter vegetation around Three Gorges reservoir. The riparian shelter vegetation construction, as one of the eco-environmental protection programmes in Post-Construction Planning of Three Gorges Project, has been conducted for many years and will be continued for some years more in the future. Many researches have demonstrated that vegetation plays an important role in the shore protection of water bodies such as rivers, lakes, and seas^8^,^9^,^10^,^11^,^12^,^13^,^14^,^15^,^16^,^17^. And, it is also found that the species composition of riparian vegetation affects the protection efficacy of riparian area by vegetation^14^,^18^,^19^. Three Gorges reservoir has shoreline of more than 5000 km and possesses vast area of riparian zone, therefore, among many candidate plants, plants which are adaptive to riparian environments, able to grow well in infertile soils and tolerate flooding are selected first and applied to construct riparian shelter vegetation. Moreover, because the riparian zone of Three Gorges reservoir has a vast area and it is laborious and also costly to construct riparian shelter vegetation over the entire riparian zone in high planting density, it is expected that all plant species in riparian shelter vegetation can spontaneously expand their populations and colonize new habitats so that the riparian shelter vegetation cover is able to gradually increase and the riparian zone of Three Gorges reservoir can be well protected.

*Salix variegata* Franch, an evergreen shrub species of Salicaceae, originally grows at riversides of Yangtze River within Three Gorges reservoir region. The species has well-developed deep roots and elegant crown, capable of tolerating inundation, growing well in barren gravel riverbanks, and withstanding scouring of river water flow. Due to the Three Gorges dam building and the subsequent rise of water level, *S. variegata* lost its original habitats at the riversides of Yangtze River. According to the programme for biodiversity conservation in Three Gorges reservoir region, it needs to be conserved *ex situ*. Because of its merits and adaptability to riparian environments, *S. variegata* has been applied to construct riparian shelter vegetation in Three Gorges reservoir region. The application of *S. variegata* in riparian shelter vegetation, concomitantly, is also beneficial to the conservation of the species. In our field observation on *S. variegata* since its application in the riparian shelter vegetation of Three Gorges reservoir, we have found that *S. variegata* in the riparian shelter belt of Three Gorges reservoir flowers and seeds every year from September to December, and produces a large number of seeds during this long reproductive season. However, only a very small quantity of *S. variegata* seedlings are produced every year in the riparian shelter belt, and in some years nearly no seedlings can be found. Owing to the small quantity of seedlings produced, *S. variegata* population has been growing very slowly in riparian shelter belt, and is unable to fast expand *S. variegata* stand to a large area, which actually prevents *S. variegata* from playing its good role in the riparian protection of Three Gorges reservoir. How to have more *S. variegata* seedlings generated to enlarge *S. variegata* population in the riparian shelter belt is of much significance for the riparian protection of Three Gorges reservoir and for the conservation of the species as well.

Basically, whether a seed can germinate to be a seedling is primarily determined by the quality and the viability of the seed^20^,^21^,^22^,^23^. If seeds produced by a plant are in poor quality or sterile, these seeds are unable to germinate and few seedlings can be produced consequently. On the other hand, if seeds are not sterile when they mature on their mother plants, but their viability can only be retained for a short time, the chance of seeds to germinate into seedlings is also low unless the seeds are able to complete germination in this short time. To unravel why *S. variegata* in the riparian shelter belt of Three Gorges reservoir produces a lot of seeds every year but does not accordingly produce numerous seedlings, we investigated the seed viability and seed germination of *S. variegata* growing in Three Gorges reservoir region. In this paper, we aim to answer the following questions: (1) Are seeds produced in *S. variegata* plants sterile? (2) Do *S. variegata* seeds retain their viability in a short time period and lose viability rapidly? (3) During the long reproductive season of *S. variegata*, do seeds produced at different time have different viability? The answers to the above-asked questions will reveal why *S. variegata* has low seedling production in the riparian shelter belt of Three Gorges reservoir, which is helpful to promote the riparian protection by *S. variegata* in Three Gorges reservoir region and the conservation of the species. Furthermore, the study on *S. variegata*, as a model species of genus *Salix*, will deepen our understanding of why many *Salix* species produce a lot of seeds but only obtain a small quantity of seedlings and why many *Salix* species have much smaller quantity of sexual descendants than their asexual descendants.

## Results

### Seed sojourn time

**Figure 1:**
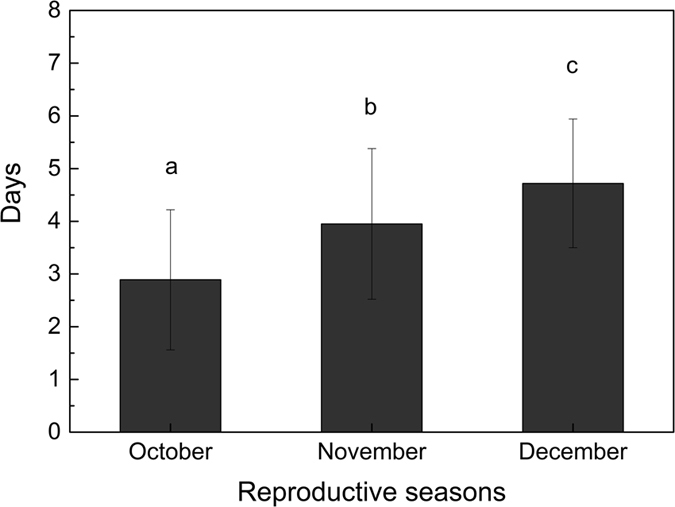
Sojourn time (mean ± sd) of mature seeds in capsules of *Salix variegata* in early, mid, and late reproductive season (i.e. October, November, and December, respectively) in Three Gorges reservoir region.

We found that whenever the seeds of *S. variegata* were produced, in October, November, or December, they stayed some days in capsules to wait for the complete dehiscence of capsules and then protruded out from the dehisced capsules. On average, the sojourn time of seeds produced in early October, mid November, and late December were 2.89 days, 3.95 days, and 4.72 days, respectively (Fig. 1). Seeds produced in late reproductive season tended to have longer sojourn time in capsules than seeds produced in early reproductive season (Fig. 1).

### Seed germination

**Figure 2:**
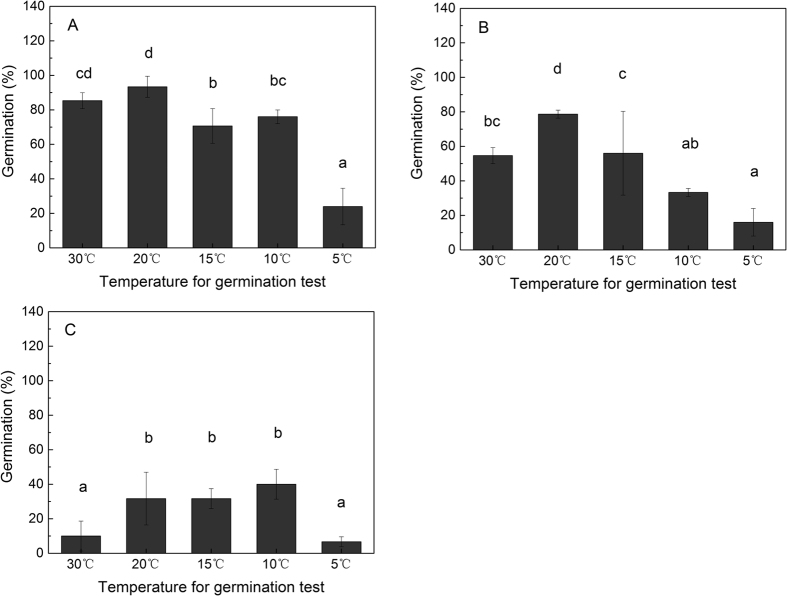
Germination rates (mean ± sd) of non-stored fresh *Salix variegata* seeds produced in early, mid, and late reproductive season (**A,B**, and **C,** respectively). The germination of seeds at temperature of 5, 10, 15, 20, and 30 °C were examined respectively.

It is found that *S. variegata* seeds produced in different reproductive seasons had different viability (indicated by germination rate). The seeds produced in October and December exhibited the highest and lowest germination, respectively. The maximal germination rates of seeds produced in October, November, and December were 93.33%, 78.67%, and 40%, respectively (Fig. 2). Furthermore, the results showed that the optimal temperature for germination was dissimilar for seeds produced in different reproductive seasons. Seeds produced in October achieved the largest germination at 30 °C and 20 °C, while seeds produced in November showed the largest germination at 20 °C, and seeds in December tended to have the largest germination at 10 °C (Fig. 2).

### Seed viability-retaining time

**Figure 3:**
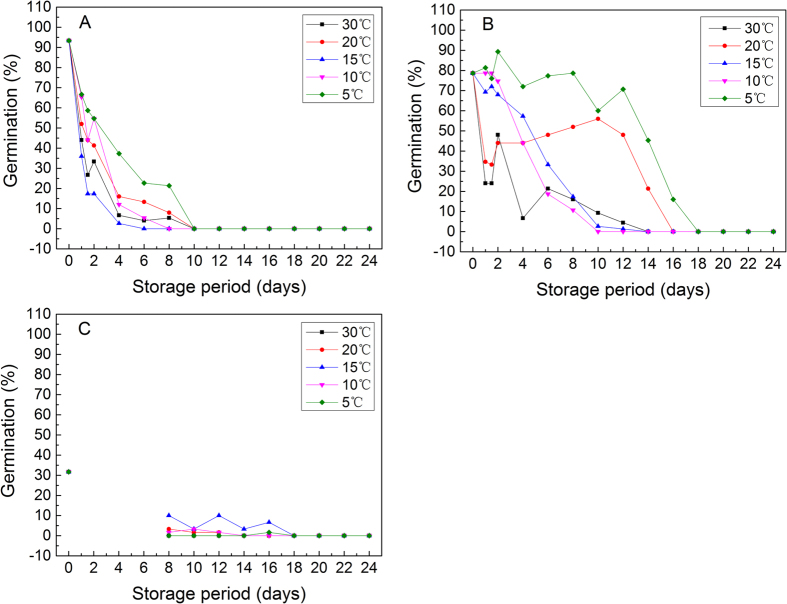
Germination performance of *Salix variegata* seeds produced in early, mid, and late reproductive season (**A,B**, and **C**, respectively). The seeds were stored for different time periods under the temperature condition of 5, 10, 15, 20, and 30 °C, respectively.

This study showed that the longest viability-retaining time of *S. variegata* seeds produced in October, November, and December was 8 days, 16 days, and 16 days, respectively (Fig. 3). Although the seeds produced in December seemed to have the longest viability-retaining time compared with seeds produced in October and November, in reality, seeds produced in December had low viability and kept a germination rate of no more than 10% after 8 days storage (Fig. 3C). For seeds produced in October, seeds stored at 30 °C, 20 °C, and 15 °C dropped their viability by nearly 50% after only 1 day (24 hours), and seeds stored at all temperatures decreased their viability to a level of less than 40% after being stored 4 days (96 hours) (Fig. 3A). For seeds produced in November, seeds stored at 30 °C, 20 °C had germination rates lower than 40% after only 1day, seeds stored 6 days at all other temperatures except 20 °C and 5 °C had germination rates lower than 35% (Fig. 3B). Generally, with respect to seeds produced in October and November, seeds stored at 5 °C decreased their viability in a relatively slower rate compared to seeds stored under other temperature conditions. However, with regard to seeds produced in December, seeds stored at 5 °C did not exhibit higher viability, at least for seeds stored for a period of longer than 8 days (Fig. 3C).

## Discussion

Although many factors may affect the germination of seeds^24^,^25^,^26^,^27^,^28^,^29^,^30^,^31^,^32^, it is undoubted that seed viability is a critical precondition determining whether a seed is able to germinate to be a seedling^22^,^23^,^25^,^33^. If seeds produced by plants of a species are in poor quality, it is difficult for the plants to successfully produce seedlings, and consequently, the population growth and persistence of the species cannot be realized easily^24^,^34^,^35^,^36^,^37^,^38^,^39^,^40^. Some studies have demonstrated that the weak population growth and spatial colonization of *Phragmites australis, Ruppia maritima, Zostera marina* can be attributed to the low viability of seeds^36^,^39^,^41^. In our study, as soon as the seeds in the capsules on *S. variegata* plants matured, the seeds were collected immediately to examine their germinability. We found that the viability of the seeds produced in early, mid, and late reproductive season (namely early October, mid November, and late December, respectively) of *S. variegata* were different. The seeds produced in early reproductive season had the highest viability (germination rate 93.33%), the seeds produced in mid reproductive season ranked the second in viability (germination rate 78.67%), and the seeds matured in late reproductive season had the lowest viability (germination rate 40%) (Fig. 2). Only the seeds produced in late reproductive season showed relatively lower viability and accordingly lower germination. Considering that most seeds of *S. variegata* in the riparian shelter belt of Three Gorges reservoir were produced in early and mid reproductive season, and the seeds produced in late season (these seeds had germination rate of 40% (Fig. 2), which was not very low in fact) only took a small proportion of all seeds produced (based on our field observation and previous study^42^), as a whole, the seeds of *S. variegata* produced in the whole reproductive season had sound viability, they were not infertile and can germinate into seedlings.

**Figure 4:**
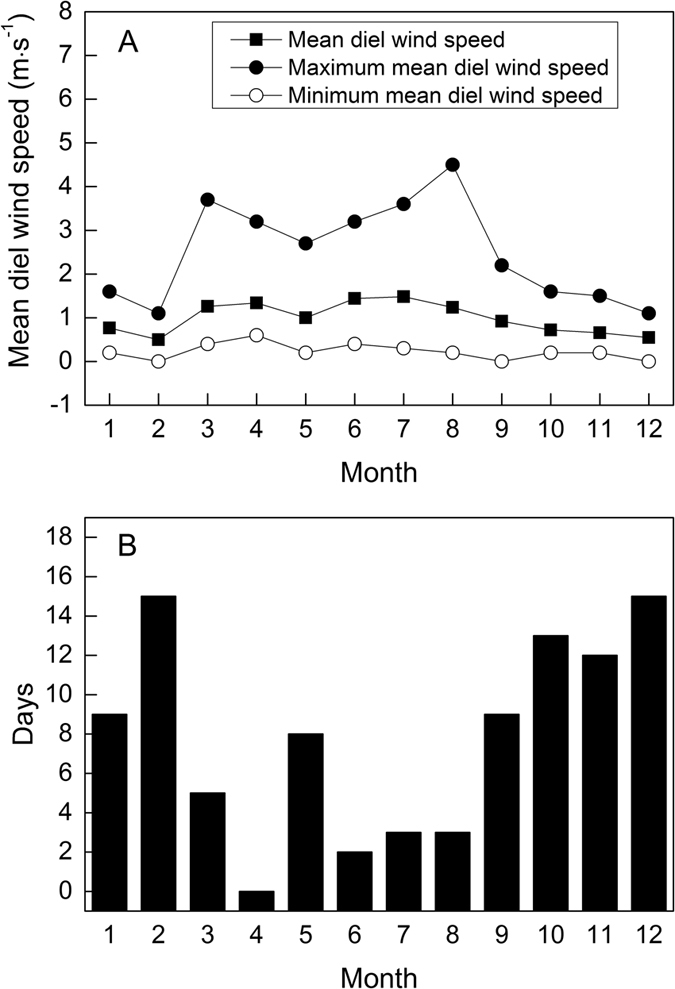
Mean diel wind speed in each month of year 2013 (**A**) and the number of days having mean diel wind speed lower than 0.6 m.s^−1^ in each month of 2013 (**B**). Data were provided by a meteorological station about 2 km away from the research station.

**Figure 5:**
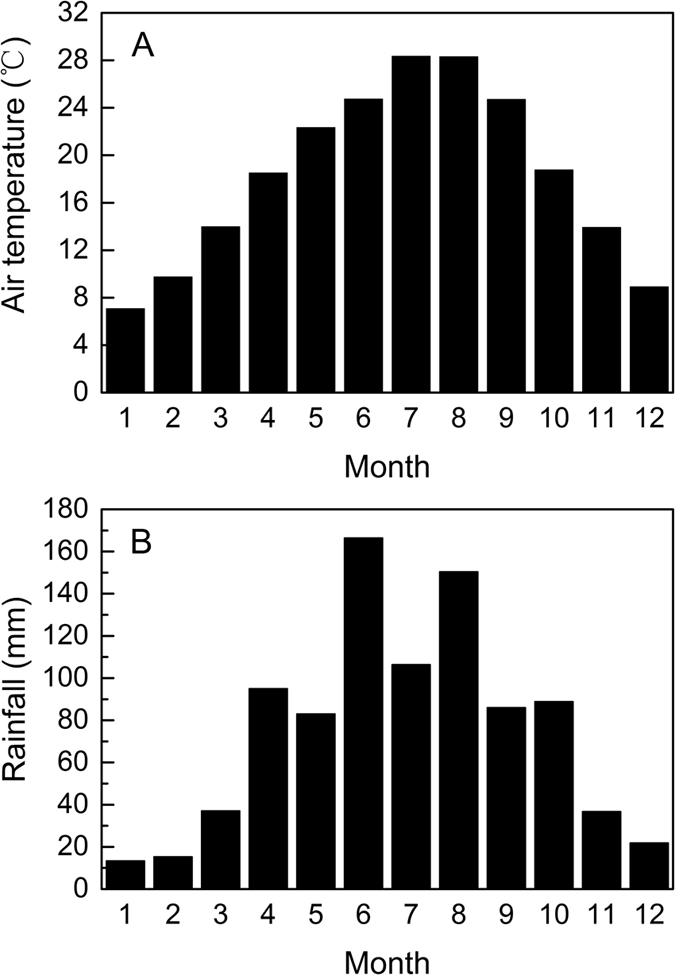
Monthly mean air temperature (**A**) and rainfall (**B**) from year 2008 to 2013 in Three Gorges reservoir region. Data were provided by a meteorological station about 2 km away from the research station.

However, in our study, it is discovered that the viability of *S. variegata* seeds decreased very quickly. Whenever the seeds were produced, in October, November, or December, whatever temperature was applied to store seeds, the retention time of seed viability did not exceed 16 days, and the seeds produced in October only retained their viability for 8 days (Fig. 3). In this study, we found that the mature seeds of *S. variegata* stayed about 3 to 5 days in capsules before they eventually emerged from the capsules, and the later the seeds were produced, the longer the seeds stayed in the capsules (Fig. 1), this implies that when *S. variegata* seeds emerged from capsules, the seed viability had already lost to a large extent. Moreover, when the tiny seeds eventually emerged from the dehisced capsules, it is very likely that the seeds can not immediately fall to the ground, but entangled with their attached silky hairs and stayed continuously on catkins if there was no wind blowing. Even if there was some wind and seeds (together with attached cotton-like hairs) were blown off the catkins, generally, it is very difficult for the tiny seeds to touch soil surface to germinate just because of the obstruction by the attached cotton-like silky hairs. Unfortunately, the reality is only weak wind or no wind occurs from October to December in Three Gorges reservoir area (Fig. 4). Therefore, in many cases, owing to the fast loss of seed viability (Fig. 3), when *S. variegata* seeds finally have chance to touch soil surface, it is probable that the viability of the seeds have already dropped to a very low level or even to zero. In addition, many studies have well demonstrated that seed germination needs sufficient and stable water supply^43^,^44^,^45^,^46^,^47^,^48^. In Three Gorges reservoir area, rainfall occurs mainly in rainy season from April to August, October to December in this area has low rainfall in a year and usually no rainfall occurs for many successive days (Fig. 5B). In the period from October to December, rainfall does not always occur to coincide with the release of *S. variegata* seeds, and damp soil surface also does not always present for seeds to germinate as long as the seeds fall to ground. Therefore, even if seeds do not stay on catkins any longer and fall to soil surface, if no rainfall occurs during the remaining viability-retaining time of these seeds, these seeds will lose the opportunity to germinate and as a result no seedlings can be produced. This is the main reason why *S. variegata* plants in riparian shelter belt of Three Gorges reservoir produce a large number of seeds every year but only have a very small number of seedlings or even no seedlings produced in some years.

Based on the germination performance of *S. variegata* seeds produced in early, mid, and late reproductive season, we found that *S. variegata* seeds produced in different reproductive seasons had dissimilar temperature preference for germination. The air temperature in Three Gorges reservoir region in early reproductive season of *S. variegata* is relatively high, the seeds produced in early season exhibited higher germination under high temperatures and lower germination under low temperatures (Fig. 2). By comparison, the air temperature in late reproductive season is relatively low, *S. variegata* seeds produced in this season exhibited higher germination under low temperatures and lower germination under high temperatures (Fig. 2). The optimal temperature for seed germination of *S. variegata* tended to correspond to the air temperature under which the seeds got mature, and the optimal temperature decreased with the drop in air temperature from October to December accordingly. Undoubtedly, this shift of optimal temperature for seed germination was of some benefit to enhance the overall seed germination of *S. variegata*. However, because the seed viability decreased very quickly and can only retain a period of less than 18 days, this shift of optimal germination temperature can not essentially increase the seedling production of *S. variegata* too much. In this study, in order to investigate the effects of temperature on the viability retention of *S. variegata* seeds, the germinability of *S. variegata* seeds stored at different temperatures was examined. It was shown that *S. variegata* seeds produced in early and mid reproductive season lost their viability much slower when stored at low temperatures than when stored at high temperatures (for instance the seeds stored at 5 °C showed the slowest viability loss) (Fig. 3A,B), this indicates that for the seeds produced in early and mid reproductive season which had good viability, storage at low temperatures was favorable to the retention of seed viability. Some studies on other *Salix* species also demonstrated that low temperature was helpful in maintaining the viability of seeds^49^,^50^,^51^, which is similar to what we found in this study. However, although low temperature was able to delay the viability loss of the seeds produced in early and mid reproductive season, because the natural air temperature in the riparian zone of Three Gorges reservoir at early and mid reproductive season of *S. variegata* is not low (usually higher than 15 °C) (Fig. 5A), therefore, low temperatures do not actually exist to delay the viability loss of the seeds produced in early and mid reproductive season. As a consequence, even though the seeds of *S. variegata* produced in early and mid reproductive season had high viability at the beginning, but due to the relatively high air temperature at that time period, these seeds lost their viability very rapidly and were unable to present high germination and high seedling production (Fig. 3A,B). In this study, unlike the seeds produced in early and mid reproductive season, the seeds produced in late reproductive season did not show high viability when stored at low temperatures, at least for the seeds stored more than 8 days (Fig. 3C). It is presumable that low temperature was not effective for decelerating viability loss of *S. variegata* seeds produced in late reproductive season.

In this study, it is elucidated why *S. variegata* in riparian shelter belt of Three Gorges reservoir produces a lot of seeds every year but does not correspondingly result in a large quantity of seedlings. Based on the experimental results in this study, we can envisage that due to the fast loss of seed viability and consequently the difficult natural generation of *S. variegata* seedlings, it is not easy for *S. variegata* to enlarge its population spontaneously in the riparian shelter belt of Three Gorges reservoir region. Therefore, to enhance the population growth and spatial exploration of *S. variegata*, some extraneous aids are needed and necessary. For instance, when seeds of *S. variegata* mature, shaking branches or blowing wind artificially can be done to accelerate seed release and reduce the sojourn time of seeds on catkins and branches. And, artificial precipitation can be conducted to facilitate the touch of seeds with soil surface and simultaneously to increase water availability for seed germination. It is well known that sexual reproduction is an important way of a plant species to maintain its genetic diversity^52^,^53^,^54^,^55^, seeds, as the outcome of sexual reproduction, can play a role in maintaining or increasing genetic diversity of a species only when they germinate into seedlings and are successfully incorporated into the population of the species. Because our study showed that *S. variegata* seeds lost their viability very quickly, therefore, in order to obtain seedlings so as to conserve the species, it is not appropriate to collect seeds after the entire emergence of seeds from dehisced capsules, instead, the suitable way is to collect seeds by pulling them out from the capsules when the capsules are just slightly dehisced, and germinate the pulled seeds immediately without any delay to produce seedlings. In addition, because the seeds produced in early and mid reproductive season showed higher viability than the seeds in late season, therefore, if the seeds of *S. variegata* are used to produce seedlings, the seeds produced in early and mid reproductive season rather than in late season ought to be used first.

In conclusion, our study shows that the seeds produced by *S. variegata* plants over the whole reproductive season in the riparian area of Three Gorges reservoir were not sterile, but in contrast had good viability. The fast loss of seed viability as well as the seed sojourn in capsules restricted the production of *S. variegata* seedling in the riparian shelter belt of Three Gorges reservoir, and consequently resulted in a slow population growth of the species. To our knowledge, this is the first demonstration that the seedling production of *Salix* plants was restricted by such a fast loss of seed viability, and which was aggravated by seed sojourn in capsules. Our findings in this study are useful in understanding why some *Salix* species produce a lot of seeds but have scarce seedlings, small populations and low genetic diversity.

## Materials and Methods

### Plant species

*Salix variegata* Franch is an evergreen shrub species of family Salicaceae. It has small leaves and elegant crown, with a height of usually 1.3–1.5 meters and 2 meters maximum. *S. variegata* is inundation-tolerant, growing originally at riverbanks of Yangtze River within Three Gorges reservoir region. It is able to grow in infertile soils and gravel riverbanks, and withstand the scouring of river water flow. *S. variegata* reproduces every year from September to December in Three Gorges reservoir region, fructifying from October to December. Every capsule on the catkins of *S. variegata* contains many small green seeds (about 1 mm in length), each having white silky hairs at the seed base. When the seeds in capsules of *S. variegata* get mature, the capsules begin to dehisce, and usually take some days to dehisce completely so that the mature green seeds and their attached white silky hairs are able to protrude out from the dehisced capsules. All protruded seeds, twisted with the white silky hairs, stay on the catkins all the time unless they are blown away by wind.

### Study area

The study was conducted in the Riparian Research Station of Southwest University, which is located in Chongqing in Three Gorges reservoir region. Three Gorges reservoir, about 665 km in length, is situated in central subtropics of China. Three Gorges reservoir region lies between 28°32′~31°44′N and 105°44′~111°39′E, the climate of this region is monsoonal, resulting in hot, humid summers, cool autumns, and chilly but mostly frost free winters (Fig. 5A). Annual precipitation at this region is more than 1000 mm, mainly falling in summers (Fig. 5B). Because Three Gorges reservoir region is situated in a basin (Sichuan Basin), the region does not have too much wind all the year round, with most wind blowing in summer (Fig. 4).

### Seed sojourn time determination

*S. variegata* in Three Gorges reservoir region have a long reproductive season and mature seeds are successively produced every year from early October to late December. In early October, mid November, and late December of 2013, which was respectively the early, mid, and late reproductive season of *S. variegata* in Three Gorges reservoir region, 100 ripe capsules on the upper crowns of approximately 30 randomly chosen *S. variegata* plants in the riparian shelter belt of Three Gorges reservoir were randomly selected and nondestructively labeled when they just began to dehisce. These 30 chosen plants scattered in an area having a shoreline length of approximately 280 m, with a distance of not less than 8 m between each two adjacent plants. All plants were growing in the riparian shelter belt at an elevation range of 175–177 m (above sea level). The time each capsule needed to dehisce completely so as to expose all inside seeds was recorded. The recorded time was regarded as the sojourn time of all mature seeds inside the capsule.

### Seed germination test

In early October, mid November, and late December of 2013, mature seeds were collected from randomly selected *S. variegata* plants in the riparian shelter belt of Three Gorges reservoir using the following procedure. These plants were growing at the same shelter belt area and elevation as the plants for seed sojourn time investigation. When capsules on catkins of *S. variegata* plants just began to dehisce, the capsules were immediately removed from the catkins, and seeds inside the capsules were released by pulling the silky hairs of seeds out of the capsules using tweezers. All collected seeds were randomly divided into two groups. Seeds of the first group were assigned to receive germination test immediately at temperature of 30 °C, 20 °C, 15 °C, 10 °C, and 5 °C respectively in climate chambers. Seeds of the second group were stored immediately in storage chambers at 30 °C, 20 °C, 15 °C, 10 °C, and 5 °C, respectively, with illumination of 12 h light/12 h dark (fluorescent light, PAR: 50 μmol.m^−2^.s^−1^). The set-up of temperature gradient was based on the air temperature in the riparian area of Three Gorges reservoir from October to December.

In the germination test for seeds from the first group, seeds of three replicates (each having 50 seeds) were put on filter paper moistened with distilled-deionized water in 9-cm-diameter Petri dishes, the filter paper were maintained moistened during the whole germination test period, all tested seeds were given illumination of 12 h light/12 h dark (fluorescent light, PAR: 50 μmol.m^−2^.s^−1^). The germination test was not terminated until all seeds had germinated or turned colour from green to black (our previous study demonstrated that seeds turned colour from green to black cannot germinate), seeds developed cotyledons, hypocotyls and roots were counted as germinable seeds.

For seeds of the second group stored under temperature conditions of 30 °C, 20 °C, 15 °C, 10 °C, and 5 °C, after different time period of storage (0, 1, 1.5, 2, 4, 6, 8, 10, 12, 14, 16, 18, 20, 22, 24 days), some stored seeds under each storage temperature were randomly sampled for germination test at 20 °C. Because the seeds produced in late December was not plentiful, and based on the germination performance of the seeds produced in October and November we supposed that the seeds produced in December had viability when stored within 8 days, therefore, the germination of December seeds stored 1, 1.5, 2, 4, 6days were not checked in the experiment. Three replicates of seeds (each having 50 seeds) were sampled for germination test, and the germination test followed the same procedure described above.

### Data analysis

In this study, germination rate of tested seeds was calculated and used in data analysis. One-way ANOVA was used to detect the effect of reproductive season on seed sojourn time and Duncan’s multiple range test was used to check the difference in seed sojourn time between reproductive seasons. Similarly, one-way ANOVA was applied to detect the temperature effect on germination of non-stored fresh seeds, Duncan’s multiple range test was used to check the difference in germination between temperatures. Data transformation was conducted to equalize variances if necessary.

## Additional Information

**How to cite this article**: Ayi, Q. *et al*. Seed sojourn and fast viability loss constrain seedling production of a prominent riparian protection plant *Salix variegata* Franch. *Sci. Rep.* **6**, 37312; doi: 10.1038/srep37312 (2016).

**Publisher's note:** Springer Nature remains neutral with regard to jurisdictional claims in published maps and institutional affiliations.
